# Reprogramming the Immune Landscape of Inflammatory Breast Cancer

**DOI:** 10.1002/advs.75766

**Published:** 2026-05-23

**Authors:** Verena Martinez‐Rodriguez, Suguru Ogata, Xiaoping Wang, Naoto T. Ueno

**Affiliations:** ^1^ Cancer Biology Program University of Hawaiʻi Cancer Center Honolulu Hawaii USA; ^2^ University of Hawaiʻi Inflammatory Breast Cancer Clinic and Research Program Honolulu Hawaii USA

**Keywords:** immune checkpoint inhibitors, immune evasion, immunotherapy, inflammatory breast cancer, programmed cell death ligand 1, translational oncology, tumor microenvironment

## Abstract

Inflammatory breast cancer (IBC) is the most aggressive subtype of breast cancer, characterized by rapid progression, early metastasis, and profound therapeutic resistance. Despite advances in multimodal therapy, outcomes remain poor, emphasizing the need for novel treatment strategies. Increasing evidence reveals that IBC exhibits an immunosuppressive tumor microenvironment (TME), driven by interactions among tumor cells and TME components that promote immune evasion through cytokine signaling loops, immune checkpoint overexpression, and formation of tumor emboli that shield cancer cells from immune surveillance. Recent multi‐omics and spatial transcriptomic studies reveal extensive immune heterogeneity, with both “immune‐hot” and “immune‐cold” niches influencing responses to immunotherapy. Clinical trials testing immune checkpoint inhibitors alone or combined with chemotherapy, antibody–drug conjugates, or targeted therapy show early promise but remain limited by small IBC cohorts and inconsistent diagnostic criteria. Integrating immune biomarkers, including PD‐L1 expression, tumor mutational burden, T‐cell clonality, and spatial immune signatures, may improve patient stratification and therapeutic precision. This review summarizes mechanistic and clinical insights defining IBC immunobiology, highlights barriers to durable immune activation, and outlines strategies to reprogram the TME toward an immunoactive state. Advancing combination immunotherapy and adaptive clinical trial designs through global collaboration will be essential to improve outcomes for IBC patients.

## Graphical abstract

Inflammatory breast cancer is the most lethal subtype of breast cancer and is characterized by an immunosuppressive tumor microenvironment (TME) driven by a complex network of immune cells and inflammatory cytokines, contributing to its aggressiveness and treatment challenges. Immune checkpoint inhibitors, either alone or in combination, show potential to counteract this immunosuppressive TME and improve patient outcomes.

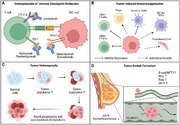

## 1 Introduction

Although recent reviews have discussed immunotherapy in inflammatory breast cancer (IBC), none have provided an extensive, mechanistically integrated analysis of the immune and stromal microenvironment in relation to therapeutic response. Prior works have primarily summarized early clinical trials and checkpoint inhibitor data, offering limited discussion of the underlying tumor–immune crosstalk or its translational implications [1, 2]. These reviews do not capture the recent advances in single‐cell and spatial transcriptomic profiling that illuminate how tumor‐associated macrophages, dendritic cells, endothelial cells, and mesenchymal stem cells (MSCs) collectively drive immune evasion and resistance in IBC. Furthermore, none have synthesized these mechanistic insights to outline how microenvironment reprogramming could enhance immunotherapy efficacy. The current review uniquely bridges cellular immunobiology with clinical trial evidence to define actionable gaps and future strategies, thereby advancing a framework to transform IBC from an immune‐refractory disease into one responsive to durable immunologic control.

### 1.1 Brief Overview of IBC

IBC is a rare subtype of locally advanced breast cancer and represents the most aggressive and lethal form of breast cancer. In the United States, IBC accounts for only 1%–5% of all breast cancer cases, with a higher incidence rate in Black patients compared to White patients [3], yet it accounts for 7%–10% of breast cancer‐related mortality due to its high metastatic rate [4]. For patients with IBC, the 5‐year overall survival rate accounts for approximately 40%. Among racial groups, White patients have a higher survival rate at 42.5%, while Black patients have a lower rate at 29.9%. Over time, survival outcomes have improved: patients diagnosed between 1978 and 1982 had an average survival of just over five years, whereas those diagnosed between 2008 and 2012 had an average survival of more than eight years [3]. Several studies have found a higher risk of developing IBC in patients with obesity [5, 6, 7]. Similar to other breast cancer types, IBC can be classified into three clinical subtypes according to the expression of the hormone receptors progesterone receptor (PR), estrogen receptor (ER), and the human epidermal growth factor receptor 2 (HER2): HER2‐positive, hormone receptor (HR) positive, and triple‐negative. In IBC, HER2‐positive or triple‐negative is more common compared to non‐IBC. At the molecular level, gene expression profiling defines intrinsic subtypes as luminal A, luminal B, HER2 overexpressing, basal‐like, and normal breast‐like, which generally correspond to, but are not identical with, the receptor‐based classification [8].

### 1.2 Limitations of Current Treatment and Immunotherapy

Because of its atypical clinical symptoms, the diagnosis of IBC remains challenging, which often leads to delayed or incorrect diagnosis. Unlike other forms of breast cancer, IBC is distinguished by the rapid onset of diffuse skin changes, including erythema and edema, known as peau d'orange, which may occur with or without a palpable mass. Operative solutions for IBC are limited due to the fast progression and rapid metastasis to nearby lymph nodes and distant organs [9].

IBC is classified as T4d in the American Joint Committee on Cancer Tumor, Node, and Metastasis system, reflecting its distinctive clinical presentation as a form of locally advanced breast cancer with increased propensity for early lymphatic and distant metastasis. Diagnosis typically relies on physical examinations, ultrasonography, mammography, and magnetic resonance imaging, followed by histopathological confirmation via core needle biopsy [10]. The recent approval of a new IBC‐specific diagnostic code by the U.S. Centers for Disease Control and Prevention is expected to improve disease registry accuracy, facilitate early treatment, and support future research efforts [11].

Despite extensive clinical efforts, no standardized treatment strategy specific to IBC has been established. Current practice generally follows subtype–based recommendations for conventional breast cancer outlined in the National Comprehensive Cancer Network Guidelines [12]. For most newly diagnosed stage III IBC, standard treatment includes neoadjuvant chemotherapy, such as anthracycline and taxane, followed by surgery and radiation. HER2‐positive IBC additionally receives targeted therapy with trastuzumab and pertuzumab, while endocrine therapy is used for HR‐positive disease. For stage IV IBC, chemotherapy combined with targeted therapy remains the standard of care. Following neoadjuvant therapy, radical mastectomy along with axillary lymph node (level I/II) dissection is strongly suggested, regardless of clinical response [13].

The overall prognosis of IBC remains poor. Although multidisciplinary management, including systemic chemotherapy, surgery, and radiotherapy, has improved clinical outcomes, therapeutic responses often differ from those of non‐IBC, and durable disease control remains difficult to achieve [14], reflecting the biological heterogeneity and therapeutic resistance of this disease.

Given these diagnostic and therapeutic challenges and the limited success of existing regimens, intensive research efforts are focused on developing novel therapeutic strategies. Immune checkpoint inhibitors (ICIs) blocking programmed cell death ligand 1 (PD‐L1) and programmed cell death protein 1 (PD‐1), such as pembrolizumab and atezolizumab (anti‐PD‐1 and anti‐PD‐L1 monoclonal antibodies, respectively), have demonstrated clinical benefit in TNBC and are being actively investigated in IBC. However, growing evidence indicates that the immunosuppressive tumor microenvironment (TME) contributes to therapeutic resistance and limits the efficacy of ICIs, providing a strong rationale for strategies aimed at reprogramming the TME. Recent studies indicate that targeting tumor‐intrinsic oncogenic signaling or inhibiting the recruitment of immunosuppressive components within the TME can enhance response to ICIs [15]. Moreover, tumor‐infiltrating lymphocytes (TILs) [16] and next‐generation immunotherapies, including chimeric antigen receptor (CAR) T‐cell therapy [17], are under evaluation as prognostic biomarkers and therapeutic targets. Collectively, these advances underscore the rapid progress in immuno‐oncology research and the potential to develop more effective therapeutic strategies for IBC.

This review explores emerging clinical and mechanistic evidence on the involvement of various immune cell types and immune checkpoints, with a focus on PD‐L1, in the pathobiology of IBC, and underscores current initiatives aimed at advancing ICI‐based immunotherapy for this highly aggressive malignancy.

## 2 Immune Landscape of IBC

Although IBC was named for its distinct phenotypic and clinical presentation, such as redness, swelling, and thickening of the skin, it is fundamentally characterized and driven by an immunosuppressive TME. This TME is enriched with distinct immune and stromal cell populations, which promote tumor growth, immune evasion, and resistance to therapy [18]. Among these cell populations, tumor‐associated macrophages (TAMs) and T cells have been studied extensively, while the roles of other components, such as endothelial cells (ECs), dendritic cells (DCs), mesenchymal stem cells (MSCs), B cells, and mast cells (MCs), are only beginning to be elucidated. The current status of understanding these immune and stromal cells in IBC tumors is summarized below.

### 2.1 TAMs

TAMs are key immune cells in the TME and are generally categorized into M1 and M2 subtypes. M1 macrophages are known to have anti‐tumor activities through direct and antibody‐mediated killing, while M2 macrophages support tumor growth by inhibiting T cell‐driven anti‐tumor responses [19]. However, recent research shows that the M1/M2 classification is overly simplistic. Macrophages are highly plastic innate immune cells that often display context‐dependent phenotypes, often exhibiting mixed features. Progress in single‐cell RNA sequencing (scRNA‐seq) and spatial transcriptomics has revealed diverse TAM subtypes with distinct functions and metabolic profiles that vary by tumor localization. Novel biomarkers, such as SPP1, MARCO, TREM2, and IDO1, help distinguish tumor‐promoting from tumor‐fighting TAMs. Studies have identified specialized TAM subsets, including inflammatory, interferon‐primed, angiogenic, immune‐regulatory, lipid‐associated, tissue‐resident, and proliferating subtypes, each characterized by unique gene signatures and metabolic adaptations. Understanding this heterogeneity is key to developing TAM‐targeted therapies and improving immunotherapy outcomes [20].

In breast cancer, TAMs drive disease progression by enhancing angiogenesis and metastasis, promoting cancer stemness, modulating cellular energy metabolism, and suppressing immune responses [21]. Notably, two populations of TAMs have recently been identified in breast cancer based on PD‐L1 expression. Breast cancer PD‐L1^+^ TAMs are more mature and exhibit immune‐stimulatory features similar to M1‐like macrophages, and tend to be located near T cells. In contrast, PD‐L1^–^ TAMs are localized near cancer cells and are less mature and immunosuppressive, like M2‐like macrophages. The presence of PD‐L1^+^ TAMs may indicate an active immune environment and may help explain better treatment outcomes to anti‐PD‐1/PD‐L1 immunotherapy in some patients [22].

In IBC, TAMs represent the most extensively studied immune cells. Immunophenotypic analyses of IBC patient samples have revealed a predominance of CD163^+^ macrophages, indicative of a highly immunosuppressive TME [23, 24]. A recent study analyzing pre‐treatment tumor samples from 161 IBC and 115 subtype‐matched non‐IBC patients further demonstrated increased infiltration of CD163^+^ TAMs in both the invasive margin and within the tumor area in IBC compared to non‐IBC. This study also showed that high TAM density is associated with inflammatory and proliferative pathways, supporting its role in promoting IBC aggressiveness. Moreover, the presence of CD163^+^ TAMS within the tumor area was associated with pathological complete response (pCR) [25].

An assessment of immune cell infiltration in tumor emboli from IBC patients showed that TAMs are among the most enriched immune cell types [23]. According to Mohamed et al. [26], their unpublished data showed that monocytes/macrophages localize around tumor emboli in IBC carcinoma tissues, influencing their orientation and behavior. In addition, this macrophage influx correlates with increased lymph node metastasis and protease activity, suggesting that cytokines and proteases secreted by TAMs may drive IBC progression.

Moreover, IBC patient blood samples collected from axillary tributaries were enriched in CD14^+^ monocytes compared to non‐IBC patient samples [24]. Isolated CD14^+^ cells from IBC patients were found to secrete significantly increased levels of TNF‐α, IL‐8, IL‐10, and MCP‐1/CCL2 than those from non‐IBC patients, enhancing IBC cell motility and invasion in vitro [27]. Valeta‐Magara et al. further uncovered an intricate autocrine–paracrine signaling loop initiated by IBC cells and involving monocytes that fosters IBC aggressiveness. They showed that IBC expresses elevated levels of monocyte chemoattractants and macrophage polarization factors, including M‐CSF, CCL2, CCL5, VEGFA, CCL3, CCL4, CCL18, CCL22, IL‐8, and CX3CL1, which differentiate monocytes into M2‐like macrophages. These M2 macrophages then secrete IL‐8 and GRO to promote IBC mesenchymal and cancer stem cell‐like phenotype via the JAK2/STAT3 pathway. The hyperactivation of IL‐8/JAK2/STAT3 signaling observed in IBC tumors underscores its clinical relevance in IBC progression [28].

AXL signaling has also been shown to regulate macrophage recruitment and polarization in IBC. Specifically, AXL signaling in IBC cells promotes the infiltration of CD206^+^ macrophages and induces polarization of monocytes toward M2 macrophages, which then secrete EREG and immunosuppressive cytokines CCL20 and CCL26 via STAT6 signaling, ultimately enhancing proliferation, migration, invasion, and tumor growth of IBC [29].

Importantly, therapeutic targeting of macrophage recruitment pathways has demonstrated efficacy in preclinical IBC models. Inhibition of the macrophage chemoattractant CSF1, and a key cytokine involved in macrophage activation, reduced M2 macrophage infiltration, delayed tumor growth, and inhibited skin invasion, characteristics of IBC [27].

In summary, these findings demonstrate that M2 macrophages are a key mediator of IBC aggressiveness. Depleting tumor‐promoting M2 macrophages, reprogramming them into tumor‐killing cells, or blocking tumor‐promoting signals that recruit them could highlight a therapeutic strategy for patients with IBC.

### 2.2 T Cells

In addition to macrophage‐driven immunosuppression, T lymphocytes also play a critical role in modulating the IBC TME. In general, activated cytotoxic T cells eliminate target cells by releasing cytolytic molecules like granulysin and granzymes, or by producing inflammatory cytokines, including TNF‐α and IFN‐γ. In cancer, persistent T cell receptor (TCR) signaling without successful cell elimination can cause T cell exhaustion ‐ characterized by high CTLA‐4, PD‐1, LAG‐3, TIM‐3, and CXCL13 expression levels. ICIs aim to prevent or reverse this exhaustion to restore T cell function [30]. In IBC, elevated expression of CTLA‐4, LAG‐3, and TIM‐3, as well as IDO and TGFβ1, suggest an exhausted T cell state in IBC [31], along with CXCL13^+^CD4^+^ T cells [32].

TILs are generally associated with improved outcomes in breast cancer, including enhanced responses to immunotherapy and more favorable prognoses. Consistent with this, immune profiling of pre‐ and post‐treatment tissues from IBC patients with stage III or *de novo* stage IV disease who were treated with neoadjuvant chemotherapy (NACT) with subsequent mastectomy revealed that TILs were enriched in tumors from patients who achieved a pCR, along with increased subsets of CD8^+^ and CD20^+^ cells. This study also demonstrated that responders exhibited increased T cell clonality and diversity, suggesting a more robust antitumor immune response [33], and thus, can serve as a dynamic biomarker for patient outcome. Another study identified a 107‐gene signature enriched for immune‐related genes that distinguished responders from nonresponders to NACT in IBC [34]. The IFN‐α and IFN‐γ pathways were hyperactivated, and the gene signature was enriched for genes involved in the CD8^+^ T lymphocyte activation process, supporting a role for TILs in response to NACT. A recent study investigated the associations between clinicopathological variables, stromal TILs (sTILs), pCR, and the prognostic value of pCR in 494 localized patients with IBC treated with NACT [35]. The study showed that the TNBC subtype has the highest TIL levels (∼10%), and higher sTILs were independently associated with pCR following NACT in the whole cohort but not in different receptor subtypes [35].

Moreover, a recent study demonstrated that higher infiltration of CD8^+^ T cells in the tumor area of IBC patients, compared to the invasive margin, was significantly associated with improved OS. However, the prognostic value was not solely driven by the absolute density of CD8^+^ T cells; rather, the ratio of CD8^+^ T cells to FOXP3^+^ regulatory T cells (Tregs) within the tumor area served as a more meaningful indicator of clinical outcome [25].

An immunophenotyping study of circulating tumor cells (CTCs) from IBC patients further supported the role of T cells in clinical outcomes. In the peripheral blood of IBC patients with CTCs, those with ≥1 or ≥5 CTCs had a reduced proportion of CD3^+^ and CD4^+^ T cells compared to patients without CTCs or with <5 CTCs, respectively. Additionally, patients with ≥1 CTCs showed decreased levels of IFN‐γ and TNF‐α‐expressing, TCR‐activated CD8^+^ T cells, along with an increased proportion of regulatory T lymphocytes compared to patients without CTCs. The combination of high CTCs and low IFN‐γ‐producing, TCR‐activated CD8^+^ T cells was associated with poor survival outcomes. Moreover, stage III and metastatic IBC patients exhibited fewer IL‐4‐producing TCR‐activated CD4^+^ T cells [36].

Together, these findings indicate that diminished T cell function and elevated CTCs correlate with poorer survival, while increased TILs and robust CD8^+^ T cell responses are associated with improved outcomes in IBC, highlighting the important role of T cells in modulating the IBC TME. Reducing or blocking Treg activity while simultaneously activating CD8^+^ T cells using checkpoint blockade, costimulatory signals, or supportive cytokines could be a promising avenue for IBC targeted therapy.

### 2.3 DCs

As professional antigen‐presenting cells, DCs play a central role in the adaptive immune response by activating T cells [37]. DCs consist of myeloid DCs (mDCs) and plasmacytoid DCs (pDCs) [38], both of which play crucial roles in innate and adaptive immunity. pDCs contribute to the innate and adaptive immune response via antigen presentation and secretion of IFN‐α [39], while mDCs contribute to the adaptive immune response via activation of cytotoxic CD8^+^ T cells and Th1 cells, through secretion of pro‐inflammatory cytokines such as IL‐6, IL‐12, and TNF‐α [40, 41]. DCs located in tumor sites, nearby lymph nodes, or distant metastases can become tolerogenic due to tumor‐derived soluble factors, exosomes, or recruited immunosuppressive cells. This suppression weakens their ability to activate effector T cells while enhancing the development of Tregs, thus enhancing cancer progression and metastasis through mechanisms such as epithelial‐mesenchymal transition (EMT) [42].

DC dysfunction has been observed in IBC. Patients with elevated CTC counts showed abnormalities in both pDCs and mDCs, as well as decreased TNF‐α and IL‐12 secretion, leading to impaired Th1 priming and inhibited anti‐tumor responses [36]. More research needs to be conducted to elucidate the precise role of DC in contributing to the immunosuppressive TME.

Promising studies suggest that restoring DC function may have therapeutic potential. Vaccinating mice with DCs transfected with FOXP3 mRNA induced FOXP3‐specific T cell responses, enhancing anti‐tumor immunity by targeting immunosuppressive Tregs. These engineered DCs also stimulate cytotoxic T lymphocytes that recognize and kill FOXP3‐expressing IBC cells [43, 44].

In summary, DC dysfunction contributes to immune evasion in IBC, and DC‐based immunotherapies may represent a promising avenue for future treatment strategies for patients with IBC.

### 2.4 ECs

ECs in the breast cancer TME contribute to tumor vascularization, growth, metastasis, and immune interactions through active participation in angiogenesis and lymphangiogenesis [45, 46]. A central mediator of these processes is VEGF, which not only drives angiogenesis but also shapes the immune landscape by regulating immune cell recruitment and phenotype via VEGFR2 signaling [47]. VEGF promotes an immunosuppressive TME by directing DCs, myeloid‐derived suppressor cells (MDSCs), and TAMs toward immunosuppressive phenotypes that support Treg accumulation and suppress T cell activity [48]. Importantly, VEGF blockade not only disrupts angiogenesis but also enhances lymphocyte infiltration and downregulates PD‐L1 on myeloid cells, thereby fostering a more immune‐permissive TME. These findings support the synergistic potential of VEGF‐targeted therapies and immunotherapies to enhance antitumor efficacy [47, 48].

In IBC, ECs exhibit particularly active involvement in angiogenesis and vasculogenesis. IBC tumor specimens exhibited elevated expression of angiogenic factors, but not lymphangiogenic factors, and increased recruitment of endothelial and endothelial precursor cells in tumor‐associated stroma, suggesting an active role of ECs in angiogenesis and vasculogenesis compared to non‐IBC tumors. Blocking VEGF and angiopoietin pathways using soluble Flt‐1 and Tie2 constructs inhibited IBC tumor growth [49].

Another study evaluated angiogenesis in IBC biopsies prior to chemotherapy and showed significantly higher vascular density and endothelial cell proliferation (ECP) index than non‐IBC tumors [50]. This discovery was further confirmed by another study showing elevated lymphatic ECP (LECP) in IBC compared with non‐IBC and normal breast tissue. Consistently, increased expression of endothelial‐ and lymphatic‐specific markers such as CD31, D2‐40, and LYVE‐1 was detected in IBC, further highlighting the active involvement of ECs in both angiogenesis and lymphangiogenesis [51]. Functional assays have also confirmed that IBC‐secreted factors can stimulate ECP [52].

Together, these findings indicate that ECs promote the aggressive angiogenic and lymphangiogenic phenotype of IBC.

### 2.5 MSCs

MSCs are multipotent progenitor cells with the ability to differentiate into different cell types and to modulate the immune system to promote tissue repair and regeneration. MSCs can be recruited to tumors, where they interact with tumor and immune cells to immunomodulate TME, thereby promoting tumor growth and metastasis in breast cancer [53, 54].

A major source of MSCs in breast tissue is adipose‐derived stromal cells (ASCs), which originate from adipose tissue [55]. Within the breast TME, ASCs differentiate into cancer‐associated fibroblasts (CAFs), thereby promoting cancer cell proliferation, invasion, and metastasis [56]. CAFs contribute to tumor growth by secreting extracellular matrix components and signaling molecules that can modulate myeloid and lymphoid cells [57].

In IBC, MSCs play a tumor‐promoting role. In a SUM149 xenograft model, co‐injection of MSCs promoted invasion and metastasis by increasing phosphorylation of EGFR (pEGFR) [58]. MSCs not only communicate with cancer cells, but also contribute to an immunosuppressive TME by promoting the polarization of undifferentiated macrophages. When MSCs were co‐cultured with macrophages, they secreted higher levels of IL‐6, which in turn promoted the invasion, mammosphere formation, and STAT3 activation in IBC cells [27].

Collectively, these data suggest that MSCs in IBC facilitate tumor progression by activating oncogenic signaling pathways such as EGFR and by reshaping the TME (e.g., through MSC, macrophage, and IL‐6 loops) to promote tumor aggressiveness. Further investigation into MSC‐driven phenotypic changes and signaling pathways in IBC may provide new insights into targeting the stromal component in IBC.

### 2.6 B Cells and MCs

B cells and MCs have not been extensively studied in the IBC TME, but emerging evidence suggests their potential relevance to IBC tumor growth and treatment response. In breast cancer, tumor‐infiltrating B cells (TIL‐B) are correlated with improved clinical outcomes, although their precise role in tumor immunity remains unclear. Higher TIL‐B densities, particularly in HER2‐positive and TNBC subtypes, are associated with favorable long‐term survival, increased immune activity, and the presence of germinal center and antibody‐secreting B cells in tertiary lymphoid structures [59]. IBC tumor samples have shown increased numbers of memory B cells, which correlated with a higher rate of pCR [60, 61].

A recent study showed that patients whose tumors contained a higher proportion of CD79α^+^ B cells in the tumor area relative to the invasive margin had significantly worse OS. This finding suggests that increased B cell presence within the TME may be associated with poorer prognosis in IBC [25].

MCs play diverse roles in breast cancer progression, and their infiltration varies by subtype, being higher in luminal than HER2‐positive or TNBC. By releasing molecules including VEGFA, TNF, CXCL1, histamine, nitric oxide, AREG, tryptase, chymase, TGFβ, and MMPs, MCs can modulate surrounding immune cells such as CD8^+^ T cells, DCs, MDSCs, and Tregs, and shape the TME to become immunosuppressive or immunocompetent. MCs also influence tumor aggressiveness and reduce the efficacy of anti‐HER2‐therapy by activating survival pathways [62].

In IBC, tumor tissues with lower MC populations prior to NACT were significantly associated with achieving pCR, whereas the close proximity of mast cells to CD163^+^ macrophages, CD8^+^ T cells, and tumor cells in non‐pCR cases suggests that MC infiltration may contribute to poor therapeutic response [33]. Additionally, Zhang et al. reported that IBC tumors exhibited a lower abundance of activated MCs compared to non‐IBC or normal breast tissue [63].

The current understanding of B cell and MC dynamics in IBC is still evolving, and further investigation into these underexplored populations will be critical for clarifying their mechanistic roles and therapeutic potential.

### 2.7 Summary

The immune microenvironment of IBC is uniquely shaped by a complex interplay among tumor, immune, and stromal cells that collectively sustain an immunosuppressive niche. Compared with non‐IBC, IBC exhibits distinct immunobiological features, including elevated inflammatory signaling, immune dysregulation, and extensive lymphovascular invasion, which together may contribute to its aggressive clinical behavior. Among immune components, TAMs are the most extensively studied and dominate the IBC TME with a strong M2‐like phenotype that enhances invasion, EMT, and angiogenesis through IL‐6/IL‐8–mediated STAT3 activation. Crosstalk between IBC cells and macrophages, mediated by AXL and CSF1/CSF1R signaling, establishes reinforcing cytokine loops that promote tumor aggressiveness. In contrast, TILs are often functionally exhausted and spatially excluded; however, tumors enriched with CD8^+^ and CD20^+^ cells demonstrate higher pCR rates by neoadjuvant systemic therapy, suggesting that immune activation, while rare, is clinically meaningful. DCs show impaired antigen presentation, while MSCs and ECs further amplify the immunosuppressive signaling network through pro‐inflammatory cytokines and abnormal angiogenesis. Emerging evidence on B cells and MCs indicates potential prognostic relevance, though their mechanistic roles remain underexplored. Together, these findings depict an immune ecosystem that is biologically active yet functionally paralyzed, explaining IBC's poor responsiveness to immunotherapy.

## 3 Current Status of Immunotherapy in IBC

IBC treatment remains challenging due to its aggressive nature and heterogeneous responses to therapy across molecular subtypes. A large cohort study of 527 patients with IBC showed that clinical outcomes vary significantly by subtype. HER2‑positive subtype shows better long‑term outcomes, likely due to the effectiveness of HER2‑targeted therapies. In contrast, TN‐IBC is associated with the worst prognosis, consistent with its aggressive biology and limited treatment options, while HR‐positive/HER2‐negative patients display intermediate outcomes regarding pCR [64]. These results confirm the substantial heterogeneity of IBC and highlight the urgent need for reliable biomarkers, including immune‐related biomarkers, to better predict therapeutic responses and guide treatment strategies for patients with IBC.

### 3.1 Immune Biomarkers for IBC Therapeutic Response

Biomarkers are biological indicators used to predict responses to treatments, including immunotherapy, which will help guide treatment selection and monitor therapeutic efficacy. In breast cancer, tumor mutational burden (TMB), PD‐L1 expression, and immune‐related gene signatures have shown potential to stratify patients for immunotherapy. Research on predictive biomarkers for immunotherapy in IBC is progressing, yet validated clinical markers specific to IBC remain limited due to its rarity and unique biology.

PD‐L1 has been investigated as a predictive biomarker for treatment response to chemotherapy and ICI in IBC. PD‐L1 expression may correlate with favorable features such as increased TILs, response to chemotherapy, and improved OS when considered alongside other immune markers [60, 61]. A study of 221 pretreatment IBC biopsies showed that patients with PD‐L1^+^ tumor cells and a high presence of CD20^+^ TILs had longer disease‐free survival (DFS) and improved breast cancer‐specific survival compared to patients without both markers, or those with only CD20^+^ TILs or PD‐L1^+^ TILs, confirming the prognostic role of PD‐L1 in combination with TILs [61]. Similarly, Van Berckelaer et al. reported PD‐L1 positivity on 42.9% of immune cells, which is higher than in non‐IBC, and demonstrated its correlation with stromal TILs and pCR [65]. However, the predictive value of PD‑L1 remains unsettled. He et al. showed that PD‐L1 expression is significantly associated with worse OS in IBC patients [66], contradicting the role of PD‐L1 as a favorable predictive biomarker in IBC. These discrepancies likely reflect differences in PD‐L1 detection methods and scoring systems across studies, which preclude a uniform cutoff and complicate the interpretation of PD‐L1's predictive value.

Beyond PD‐L1, other molecules emerge as potential immune‐related biomarkers for ICI response. In a clinical study investigating the anti‐PD‐1 monoclonal antibody pembrolizumab, patients with high baseline T‐cell clonality exhibited significantly longer progression‐free survival (PFS) (10.4 vs. 3.6 months) compared to those with low clonality. Patients who achieved stable disease also showed greater increases in T‐cell clonality during treatment (20% vs. 5.9%). These results suggest that T‐cell clonality may serve as a predictive marker for better response to immunotherapy [67].

TMB has correlated with ICI sensitivity in some cancer types. However, in breast cancer, and particularly in IBC, TMB is typically low to intermediate, limiting its predictive value [68]. Conversely, immune‐related gene signatures, such as IFN‐γ signatures, and the spatial distributions of TILs are promising predictive biomarkers [69, 70], highlighting the importance of multivariate predictive models that integrate multiple biomarkers rather than relying on a single one.

A transcriptomic analysis of 137 IBC and 252 non‐IBC tumors from the World IBC Consortium further demonstrated that IBC tumors overexpress various actionable immune genes (*CD274/PDL1*, *CTLA4, LAG3*, *IDO1*, *HAVCR2/TIM3*, *CD27*, *CD70*, *TNFRSF9*, *ICOS*, *PDCD1*, *PVRIG*, and *TIGIT*). Overexpression of these genes was correlated with better pathological responses to chemotherapy and suggests potential sensitivity to ICIs [60].

Together, these findings indicate the need for future clinical trials to incorporate multidimensional biomarker assessments combining PD‐L1, TMB, RNA‐based immune signatures, and spatial immune profiling to eventually improve the prediction of ICI responses in IBC patients.

### 3.2 ICIs

Given the high expression of immune checkpoint molecules in IBC tumors [31, 66], ICIs have been considered as treatment options. Recent studies indicate that ICIs primarily targeting PD‐1/PD‐L1 have introduced new therapeutic opportunities within the conventional tri‐modality treatment paradigm of chemotherapy, surgery, and radiotherapy [60]. However, clinical trials specifically in IBC remain limited, and large‐scale randomized trials focusing solely on IBC are scarce [71]. IBC patients are often included as part of broader breast cancer cohorts in immunotherapy trials, which primarily aim to investigate the efficacy and safety of ICIs and to identify predictive biomarkers.

Several ongoing studies explicitly including IBC, such as the phase II clinical trial of pembrolizumab (NCT02971748), have been conducted; however, the small sample sizes and variability in clinical diagnosis make it difficult to draw IBC‐specific conclusions. The key challenges in advancing IBC immunotherapy include: (i) the rarity of IBC, which limits statistical power in dedicated cohorts; (ii) heterogeneity in patient identification and baseline characteristics due to reliance on clinical diagnosis; and (iii) the predominance of sub‐analyses within general breast cancer trials, limiting the assessment of efficacy and toxicity outcomes. A summary of current ICI‐related clinical trials that include IBC is presented in Table 1.

**TABLE 1:** Summary of clinical trials including immunotherapy in patients with IBC.

Agents	Patient population	Mechanism of action	Phase	ClinicalTrials.gov identifier	Status
Nivolumab (anti PD‐1 mAB^a^) with NACT^b^	All IBC subtypes	Inhibition of T cell suppression and activation of apoptotic pathways	II	NCT03742986	Completed
Atezolizumab (anti‐PD‐L1 mAB), cobimetinib (MEK inhibitor), and eribulin (microtubule inhibitor)	Chemotherapy‐resistant metastatic IBC	Inhibition of T cell suppression, inhibition of the ERK signaling pathway, and induction of cell apoptosis	II	NCT03202316	Active, not recruiting
Pembrolizumab (anti‐PD‐1 mAB) combined with standard chemotherapy	HER2‐negative IBC	Inhibition of T cell suppression and inhibition of the cell cycle	II	NCT03515798	Completed
Pembrolizumab	HR‐positive IBC	Inhibition of T cell suppression	II	NCT02971748	Active, not recruiting
CAR‐macrophages CT‐0508 (anti‐HER2) with or without pembrolizumab	HER2‐overexpressing solid tumors	Inhibition of T cell suppression and activation of M1‐like macrophages	I	NCT04660929	Active, not recruiting
Pembrolizumab and oral metronomic cyclophosphamide (nitrogen mustard drug)	Locally recurrent, inoperable, and/or metastatic IBC with lymphangitic spread to the chest wall	Inhibition of T cell suppression, interfering with DNA replication and transcription processes	II	NCT03971045	Recruiting
Investigational drug combination trastuzumab deruxtecan (HER2‐ADC^c^) and durvalumab (anti‐PD‐L1 mAB)	Stage III, HER2‐positive or HER2‐low IBC	Inhibition of T cell suppression, HER2‐mediated cell death	II	NCT05795101	Recruiting
Bemcentinib (AXL inhibitor) combined with pembrolizumab	Locally advanced and unresectable, or metastatic TNBC or TN‐IBC	Inhibition of T cell suppression, inhibition of the AXL signaling pathway	II	NCT03184558	Terminated
Nivolumab and Ipilimumab (anti‐CTLA‐4 mAB)	Metastatic recurrent HER2‐negative IBC	Inhibition of T cell suppression	II	NCT02892734	Terminated
Panitumumab (anti‐EGFR mAB) and Pembrolizumab combined with NACT	Stage III‐IV TN‐IBC	Inhibition of T cell suppression, inhibition of EGFR signaling pathway	II	NCT05177796	Withdrawn
Pembrolizumab	Stage IV metastatic or recurrent IBC or non‐IBC TNBC	Inhibition of T cell suppression	II	NCT02411656	Active, not recruiting

^a^ mAB: monoclonal antibody.

^b^ NACT: neoadjuvant chemotherapy.

^c^ ADC: antibody–drug conjugate.

### 3.3 ICIs in Combination Treatments

Most clinical trials in IBC investigate the efficacy of ICIs in combination with FDA‐approved chemotherapies or targeted therapies.

A multicenter, multicohort phase II trial with an open‐label design (NCT03742986) enrolled 52 newly diagnosed patients with non‐metastatic IBC of all subtypes to test nivolumab (anti‐PD‐1) in combination with chemotherapy. The HER2‐negative cohort received nivolumab with paclitaxel followed by doxorubicin and cyclophosphamide (AC), whereas the HER2‐positive cohort received nivolumab with taxane, pertuzumab, and trastuzumab, followed by AC. Early results suggest that adding nivolumab enhances anti‐tumor activity and increases pCR rates, and full data are pending [72].

An ongoing phase II clinical trial (NCT03202316) investigates a multi‐agent regimen for patients with recurrent or metastatic IBC who have progressed after at least one prior chemotherapy line. The study evaluates atezolizumab (anti‐PD‐L1) combined with cobimetinib (MEK inhibitor) and eribulin (microtubule inhibitor). Patients first receive atezolizumab plus cobimetinib, followed by eribulin at standard doses to form a triple‐therapy protocol. After four cycles, patients transition to maintenance therapy with the targeted agents until disease progression or unacceptable toxicity. The trial's primary endpoint is overall response rate (ORR) [73].

Another ongoing phase II, multicenter, randomized, open‐label study (NCT03515798) is assessing the safety and efficacy of pembrolizumab (anti‐PD‐1) in combination with standard chemotherapy in untreated, HER2‐negative, non‐metastatic IBC. Patients are randomized to be given pembrolizumab intravenously (IV) every 3 weeks alongside epirubicin and cyclophosphamide (EC), followed by weekly paclitaxel, compared with standard EC and paclitaxel chemotherapy alone. The primary endpoint is the pCR rate following neoadjuvant therapy [74].

Another active phase II clinical trial (NCT02971748) assesses pembrolizumab in HR‐positive, localized IBC patients receiving hormone therapy who did not achieve a pCR after chemotherapy. This study evaluates 2‐year DFS, safety, and toxicity of pembrolizumab combined with hormonal therapy. It also explores associations between immune biomarkers (e.g., PD‐L1) and treatment outcomes. Pembrolizumab is administered by IV every 21 days for up to 24 months, with follow‐up at 1‐ and 24‐month post‐treatment.

A phase I clinical trial (NCT04660929) is investigating the effectiveness of CAR‐macrophages (CT‐0508, anti‐HER2 CAR macrophages) with or without pembrolizumab in HER2‐overexpressing tumors, including IBC. The primary outcomes focus on assessing the safety, tolerability, and manufacturing feasibility of CT‐0508, both as a standalone therapy and in combination with pembrolizumab, by analyzing adverse event frequency/severity and product release success. Secondary outcomes include ORR and PFS [75].

Currently recruiting clinical trials also include an open‐label, non‐randomized phase II trial at a single institution (NCT03971045) investigating pembrolizumab with oral metronomic cyclophosphamide in IBC patients with locally recurrent, unresectable, and/or metastatic disease with lymphangitic chest wall spread and no prior PD‐1 or CTLA‐4 therapy. Primary and secondary objectives include assessing efficacy, duration of response, time to progression, PFS, and OS [76].

Another phase II open‐label trial (NCT05795101) is evaluating the safety and efficacy of combining trastuzumab deruxtecan (T‐DXd), a HER2‐targeted antibody–drug conjugate (ADC), with durvalumab (anti–PD‐L1) as neoadjuvant therapy in patients with stage III IBC who have not received prior treatment. Participants are stratified by HER2 status and undergo baseline and on‐treatment biopsies, as well as blood collection for correlative studies. T‐DXd and durvalumab are administered intravenously once every 21 days for up to eight cycles, with dosing determined according to study protocol. The trial's primary endpoint is the pCR rate, and secondary endpoints include residual cancer burden, event‐free survival, distant progression, or distant disease‐free survival. Although both agents are FDA‐approved for other cancers, their use in IBC remains investigational, with this trial aiming to determine whether dual targeting of HER2 and PD‐L1 can improve outcomes in this aggressive disease [77].

A multi‐center phase II study (NCT03184558) evaluated bemcentinib (AXL inhibitor) plus pembrolizumab in previously treated patients with locally advanced, unresectable, or metastatic TNBC or TN‐IBC, with ORR as its primary objective. Participants received a dose of bemcentinib at 400 mg on days 1–3, and an IV infusion of pembrolizumab every 3 weeks starting on day 1. From day 4 onward, participants continued daily bemcentinib at a dose of 200 mg and pembrolizumab every 3 weeks until disease progression, unacceptable toxicity, consent withdrawal, or completion of 106 weeks. This trial was terminated due to lack of efficacy, underscoring the need for rational, biomarker‐guided strategies [78].

Another phase II trial (NCT02892734) assessed nivolumab (anti‐PD‐1) plus ipilimumab (anti‐CTLA‐4) in metastatic recurrent HER2‐negative IBC, a regimen approved for advanced melanoma. The trial's primary endpoint was to assess PFS, with secondary objectives to assess ORR, clinical benefit rate, OS, safety, and tolerability. The treatment regimen included administration of nivolumab via IV over 30 min every 2 weeks, and ipilimumab via IV over 90 min every 6 weeks until disease progression or unacceptable toxicity developed. This study was terminated early due to slow patient accrual, therefore limiting meaningful evaluation of efficacy.

A phase II clinical trial (NCT05177796) investigating panitumumab and pembrolizumab with NACT in patients with newly diagnosed stage III‐IV TN‐IBC was withdrawn before enrollment. The planned regimen would have included a 7‐day (cycle 0) regimen of pembrolizumab and panitumumab, followed by four 21‐day cycles (cycles 1–4) with panitumumab, pembrolizumab, paclitaxel, and carboplatin on specific days. If tolerated, patients would proceed to cycles 5–8, receiving standard care with pembrolizumab, doxorubicin, and cyclophosphamide every 21 days. The planned primary outcome was pCR.

An international retrospective cohort study evaluated the effectiveness of ICIs in combination with first‐line chemotherapy in 41 patients with metastatic TN‐IBC from eight international IBC referral centers. ICIs plus first‐line chemotherapy were administered to all patients, and 24% also had breast surgery and/or radiotherapy. Despite promising preclinical data, the 6‐month PFS rate was only 30%, with a median OS of 15.7 months, indicating limited benefit from ICIs in aggressive cancer subtypes [79].

In summary, these clinical trials highlight several important challenges in developing immunotherapy for IBC. The rarity, aggressiveness, and poor prognosis of IBC result in difficulties in patient recruitment, leading to early termination or limited statistical power. In addition, improved patient selection and biomarker‐driven strategies may be necessary. Future clinical trials in IBC may benefit from biomarker‐based patient stratification, rational combination therapies, and multi‐institutional trial designs to improve patient enrollment and therapeutic efficacy.

### 3.4 ICIs as Monotherapy

Immunotherapy as monotherapy has also been tested in IBC, but clinical data remained limited. An ongoing phase II clinical trial (NCT02411656) is investigating the efficacy of pembrolizumab as maintenance therapy in stage IV metastatic IBC or TNBC patients after achieving clinical response or stable disease to systemic chemotherapy. Patients receive pembrolizumab via IV every 21 days for 8 cycles, followed by an increased dose every 42 days for up to 24 months, unless disease progresses or side effects become unacceptable. After completing treatment, follow‐up visits are scheduled at approximately 1 and 3 months. Results showed that patients with higher baseline T‐cell clonality had improved PFS (10.4 months) compared to those with lower clonality (3.6 months). Moreover, patients whose disease remained stable during treatment showed a greater increase in T‐cell clonality (about 20%) than those whose disease progressed (about 5.9%), indicating that pembrolizumab monotherapy is effective in disease control [67].

### 3.5 Summary

Immunotherapy for IBC remains in its early translational phase, with encouraging biological rationale but limited clinical validation. IBC tumors exhibit higher PD‐L1 expression and immune gene activation than non‐IBC, suggesting an immunogenic potential; however, the predictive value of PD‐L1 remains inconsistent across studies, reflecting assay heterogeneity and the complex immune landscape of IBC. While exploratory biomarkers such as T‐cell clonality, immune‐related gene signatures, and spatial immune profiles show promise, none have been clinically validated to guide immunotherapy in IBC. TMB is generally low to intermediate, further limiting its predictive value. Collectively, these findings highlight the urgent need for multidimensional biomarker frameworks that integrate PD‐L1 expression, TMB, immune gene signatures, and spatial context to refine patient selection and therapeutic monitoring.

The clinical application of ICIs in IBC has largely been extrapolated from broader breast cancer trials. Most ongoing studies investigate PD‐1/PD‐L1 blockade, typically pembrolizumab, nivolumab, or atezolizumab in combination with chemotherapy, targeted agents, or ADCs. The emergence of CAR‐macrophage and macrophage–targeted approaches (e.g., CT‐0508 ± pembrolizumab, NCT04660929) also represents a novel avenue to remodel the immunosuppressive TME. Early results suggest that adding ICIs to neoadjuvant or metastatic treatment regimens may improve pCR and PFS, but IBC‐specific conclusions remain premature due to small sample sizes, inconsistent diagnostic criteria, and limited statistical power. The rarity of IBC and heterogeneous clinical presentation continues to constrain trial enrollment and generalizability. Encouragingly, recent trials such as NCT03742986 (nivolumab + chemotherapy) and NCT05795101 (T‐DXd + durvalumab) demonstrate growing global interest in designing IBC‐focused immunotherapy studies. The establishment of a new diagnosis code for IBC is expected to improve medical record keeping, facilitate accurate care coordination, and enhance clinical research efforts for IBC patients. This advancement will accelerate multi‐center collaboration and the design of IBC‐specific clinical trials, ultimately advancing effective immunotherapy strategies for patients with IBC.

## 4 Mechanism of Immune Evasion of IBC Cells

**FIGURE 1:**
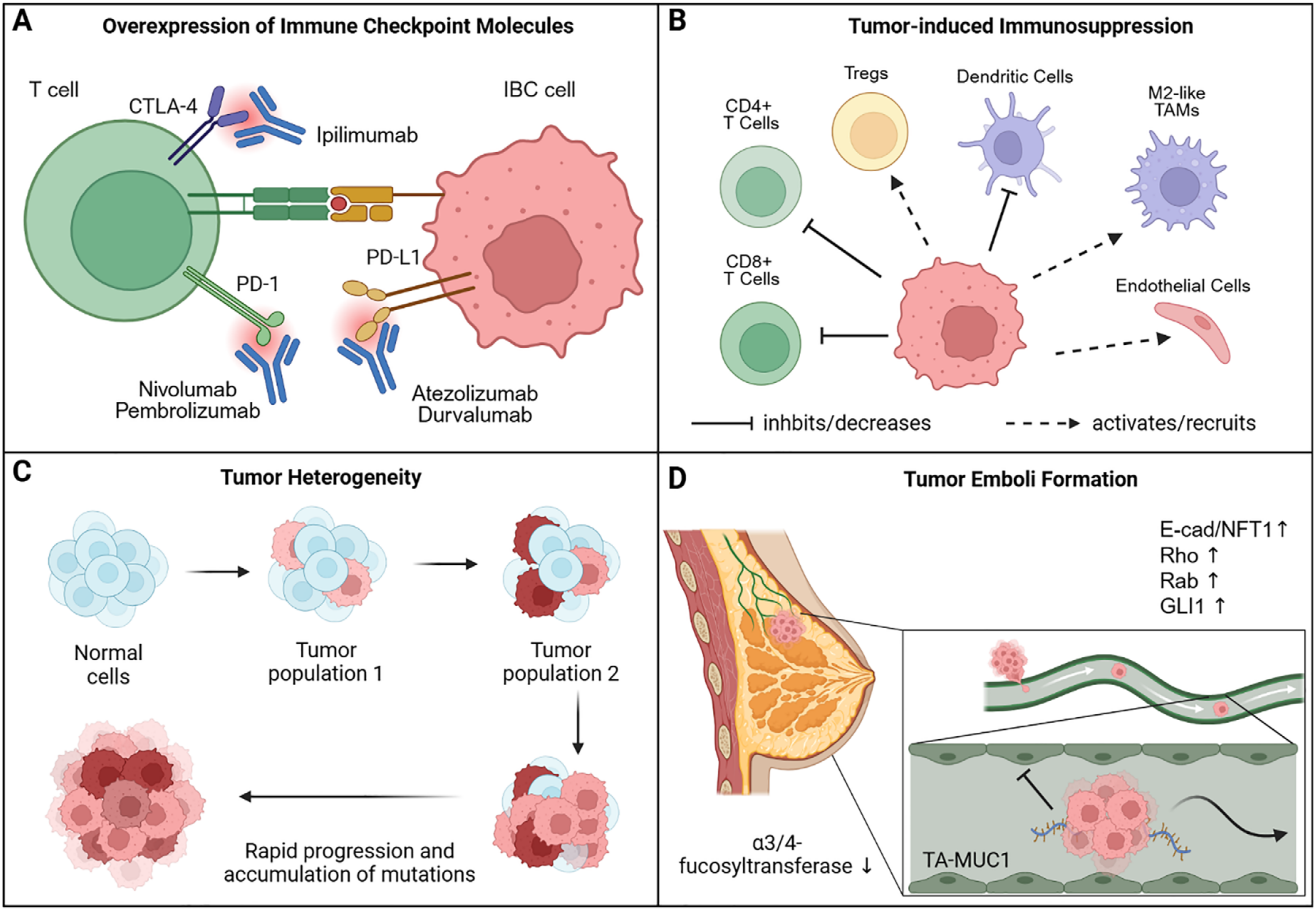
Mechanisms of immune evasion in IBC. (A) IBC cells overexpress PD‐L1, resulting in T‐cell exhaustion and suppression of cytotoxic immune responses. Current ICIs in clinical trials include the anti‐PD‐1 mABs nivolumab and pembrolizumab, the anti‐PD‐L1 mABs atezolizumab and durvalumab, and the anti‐CTLA‐4 mAB ipilimumab. (B) IBC fosters an immunosuppressive microenvironment enriched with Tregs and M2‐like TAMs while reducing cytotoxic CD8^+^ and helper CD4^+^ T cells. Recruitment and differentiation of monocytes into M2 macrophages, impaired dendritic cell function, and pro‐angiogenic endothelial activity further enhance immune suppression. (C) Heterogeneity in IBC enables immune evasion by creating phenotypically diverse tumor cells and spatially heterogeneous immune microenvironments that protect tumor regions from immune attack. (D) Tumor emboli, a hallmark of IBC, contribute to immune evasion. E‐cadherin overexpression supports cohesive cell–cell adhesion and collective dissemination, while E‐cadherin cleavage (E‐cad/NFT1, regulated by Rab7) and altered mucin glycosylation (TA‐MUC1) reduce vascular adhesion and promote embolus spread. GLI1 activation in TN‐IBC promotes proliferation, motility, tumor growth, and embolus formation. Figure created with BioRender.

Although ICIs have revolutionized the therapeutic landscape of breast cancer, especially clinical trials in TNBC patients, including IBC, have shown only moderate sensitivity to immunotherapy. Tumor immune evasion represents a major barrier to effective immunotherapy for cancer patients, including those with breast cancer. Emerging evidence indicates that tumor cells develop multiple immune evasion mechanisms, including upregulation of immune checkpoint molecules that inhibit cytotoxic T cell activity, reshaping of the TME by recruiting immunosuppressive cells through tumor intrinsic effects, secretion of immunosuppressive cytokines and chemokines, downregulation of antigen presentation machinery such as MHC class I, and metabolic alterations. IBC exhibits distinct mechanisms of immune evasion, contributing to its rapid progression and resistance to therapy (Figure 1: Mechanisms of immune evasion in IBC and current ICIs in clinical trials).

### 4.1 Overexpression of Immune Checkpoint Molecules and T Cell Exhaustion

One key mechanism of immune evasion in IBC involves the overexpression of immune checkpoint molecules, including PD‐L1, which interacts with PD‐1 on T cells, and therefore dampens the adaptive immune response [80] (Figure 1A). Several studies have shown the high PD‐L1 expression in IBC. In a cohort of 68 IBC patients, PD‐L1 expression was found in 25 cases [66]. In another large cohort study of 112 IBC and 194 non‐IBC samples, PD‐L1 expression was increased in IBC compared to non‐IBC samples; 38% of IBC samples exhibited PD‐L1 upregulation. PD‐L1 expression was associated with HER2‐enriched, ER‐negative, and basal IBC subtypes, and also correlated with better pathological response to chemotherapy [31].

High PD‐L1 expression in IBC correlated with increased levels of T cell exhaustion markers CTLA‐4, LAG‐3, and TIM‐3, along with elevated cytokines IDO and TGFβ1 that are typically expressed by exhausted T cells, suggesting an exhausted T cell phenotype in IBC TME [31].

Transcriptomic profiling of 137 IBC and 252 non‐IBC clinical samples revealed that two‐thirds of targetable immune genes (*CD274/PDL1*, *CTLA4, LAG3*, *IDO1*, *HAVCR2/TIM3*, *CD27*, *CD70*, *TNFRSF9*, *ICOS*, *PDCD1*, *PVRIG*, and *TIGIT*) were overexpressed in IBC compared to non‐IBCs [60]. These findings collectively indicate that IBC cells exploit multiple inhibitory pathways to dampen antitumor T cell responses.

Consistent with these data, immunohistochemical staining of 221 pretreatment IBC biopsies showed PD‐L1 expression in 8% of tumor cells and in 66% of TILs. Moreover, CD20^+^ TILs were present in 62% of cases, and PD‐L1‐positive tumor cells were associated with high TILs and CD20^+^ TILs, suggesting a role for B cells (CD20^+^) in anti‐tumor immune response and the progression of IBC [61]. These studies showed that PD‐L1 expression correlates with improved OS and pCR to chemotherapy [60]. Interestingly, Van Berckelaer et al. reported PD‐L1 positivity on 42.9% of immune cells, which is higher than in non‐IBC, and demonstrated its correlation with stromal TILs and pCR [58].

Taken together, these findings imply that the overexpression of immune checkpoint molecules and the resulting T cell exhaustion suppress cytotoxic immune responses, representing a mechanism of immune evasion in IBC.

### 4.2 Tumor‐Induced Immunosuppression

The major characteristics of IBC are its aggressiveness and high rate of metastasis, due to its ability to generate a highly immunosuppressive TME [18]. Molecular changes of IBC, such as overexpression of EGFR, HER2, AXL, E‐cadherin, RhoC GTPase, NF‐κB, JAK/STAT, COX‐2, angiogenic factors, and eIF4GI, loss of WISP3, and enrichment of cancer stem cell population, drive IBC progression, tumorigenesis, inflammation, and metastasis. Some of these tumor‐intrinsic features also contribute to the reprogramming of TME (Figure 1B).

#### 4.2.1 EGFR

EGFR overexpression is reported in 30% of IBC cases [81]. In a single‐arm, open‐label, phase II clinical trial (NCT01036087), the combination of panitumumab, a humanized anti‐EGFR antibody, with NACT resulted in a 42% pCR rate in TN‐IBC, suggesting that EGFR is a promising therapeutic target for IBC. The EGFR pathway regulates the cross‐talk between cancer cells and TME components in IBC. In IBC patients, pEGFR expression in the stroma correlates with its expression in tumor cells, but not in non‐IBC patients [58]. Compared to tumors grown from SUM149 cells only, those produced by co‐injecting SUM149 with MSCs showed higher expression levels of pEGFR and more metastasis, which could be abrogated by EGFR inhibitor erlotinib [58]. Further study demonstrated that EGFR‐targeted therapy remodels the TME of IBC by enhancing cytotoxic T cells while reducing immunosuppressive M2 macrophages and Tregs through suppression of EGR1‐regulated immunosuppressive chemokines. This immunoactivation of the IBC TME by an anti‐EGFR antibody improved the antitumor efficacy of an anti‐PD‐L1 antibody [82].

#### 4.2.2 X‐Linked Inhibitor of Apoptosis Protein (XIAP)

XIAP belongs to the IAP family and plays an important role in the crosstalk between the NF‐kB and MAPK pro‐inflammatory pathways. High expression of XIAP was reported in tumor samples from IBC patients. XIAP expression significantly correlates with high PD‐L1 expression and increased infiltration of TAMs and FOXP3^+^ Tregs, due to increased levels of CSF‐1 [83]. These IBC TAMs secrete TNF‐α, which binds to TNFR1 on cancer cells and activates the MAPK signaling pathway, further inducing the expression of XIAP and SOD2, and XIAP‐activated NF‐κB target genes involved in suppressing reactive oxygen species and thus, protect against cell death [83]. Moreover, a recent study showed that soluble E‐cadherin binds to XIAP and activates NF‐κB signaling, which promotes anoikis resistance and IBC cell metastasis to the brain [84].

#### 4.2.3 RhoC GTPase

The oncogenic RhoC GTPase is involved in cytoskeletal rearrangement and is essential for cell motility and focal adhesion kinetics [85, 86]. It is overexpressed in over 90% of IBC and is a driver of IBC metastasis [87, 88] and a regulator of tumor cell metabolism [89]. RhoC is required for the increased migratory activity of IBC cells to macrophage‐conditioned media (MCM) [90]. The MCM key cytokines IL‐6, IL‐10, and IL‐8 stimulate the migration of IBC cells, but not IBC cells with RhoC knocked down [90]. These findings indicate the role of RhoC GTPase in mediating the crosstalk between macrophages and tumor cells. The reciprocal effect of tumor‐derived RhoC GTPase on macrophage and other tumor immune microenvironment components is worth further investigating.

#### 4.2.4 Cytokines and Chemokines

**FIGURE 2:**
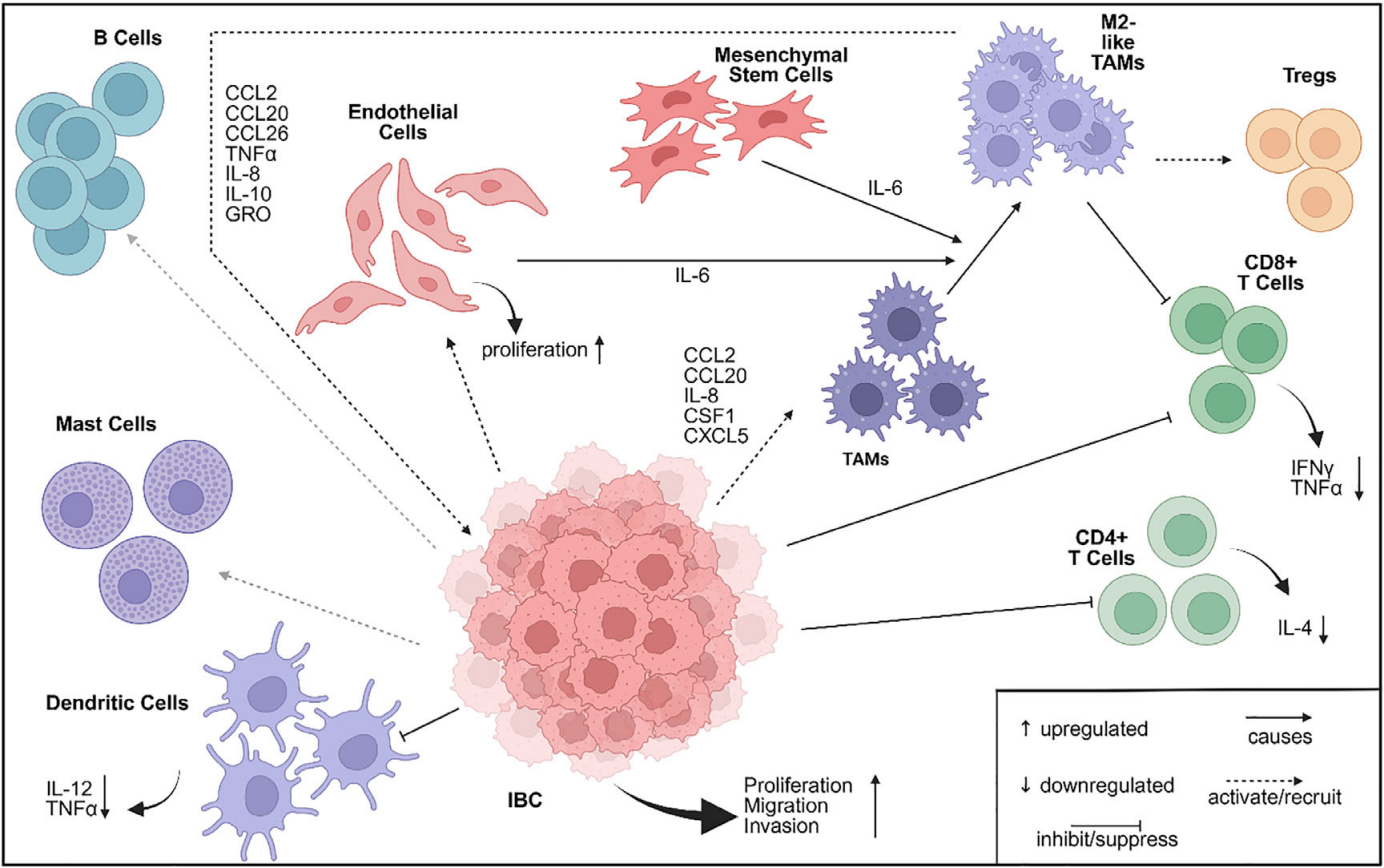
Immune landscape of IBC and the complex cytokine network. IBC cells secrete cytokines such as CCL2, CCL20, CXCL5, and IL‐8 that recruit Tregs and M2 macrophages while suppressing T‐cell cytotoxicity. Elevated CSF‐1 promotes TAM infiltration, and TAM‐derived TNF‐α protects tumor cells from apoptosis. IL‐8 and GRO enhance monocyte recruitment and M2 polarization, amplifying STAT3 signaling and driving EMT. M2 macrophages further secrete CCL20 and CCL26 to drive tumor growth and invasion. DCs and T cells show reduced cytokine production and impaired anti‐tumor responses. ECs and MSCs contribute to angiogenesis and immunosuppression via IL‐6‐mediated macrophage polarization. Figure created with BioRender.

High expression of inflammatory cytokines and chemokines, regulated by inflammatory pathways such as JAK/STAT, NF‐κB, and COX‐2, as well as oncogenic proteins including EGFR, AXL, and RhoC GTPase, is a hallmark of IBC tumors. Cytokines such as IL‐6, IL‐8, IL‐1β, CCL2, and TNF‐α are overexpressed in IBC cells and patient samples. Their secretion, either from cancer cells or stromal components, reprograms immune cells within the TME to become more tumor‐supportive (Figure 2: Complex cytokine network shapes the immunosuppressive TME in IBC) [28, 29, 82, 91].

Among these molecules, IL‐6 and IL‐8 are hyperactivated through NF‐κB regulation. IL‐6 activates JAK/STAT3 signaling, promoting tumor cell survival and EMT in IBC. M2 macrophage‐educated MSCs secrete more IL‐6, enhancing IBC invasion and mammosphere formation. Conversely, inhibiting TAM recruitment decreases the expression of pSTAT3 and secretion of IL‐6, delaying IBC tumor formation and decreasing skin invasion and local recurrence [27].

Similarly, IL‐8 contributes to immune modulation and tumor invasion in IBC. High IL‐8 expression promotes neutrophil recruitment and a pro‐tumorigenic inflammatory milieu. Valeta‐Magara et al. reported that IL‐8 and its downstream JAK2/STAT3 signaling are more highly expressed in IBC tumors than in normal breast tissues. Overexpression of IL‐8 and GRO activates JAK2/STAT3, inducing expression and the differentiation of monocytes into pro‐tumorigenic M2 macrophages. These macrophages secrete more IL‐8 and GRO, further activating STAT3 signaling to promote EMT and invasion [28]. As noted in the “RhoC GTPase” section, macrophage‐secreted IL‐6, IL‐8, and IL‐10 also stimulate IBC cell migration in a RhoC‐dependent manner [90].

Beyond IL‐6 and IL‐8, CCL2 also plays a key role in shaping the IBC TME. IBC‐derived CCL2 increases TAM recruitment and promotes tumor growth and metastasis. Knockdown of CCL2 in IBC cells reduces TAM density, suppresses tumor growth, and increases necrosis, highlighting its role in promoting IBC aggressiveness through TAM reprogramming [91]. Furthermore, EGFR/EGR1‐regulated chemokines, including CXCL5, CCL2, CCL20, and IL‐8, have been shown to recruit Tregs and TAMs while impairing CD8^+^ T‐cell infiltration [82]. An analysis of the IBC World Consortium Dataset revealed that high CCL20 expression correlates with worse OS [82].

Recent multi‐omics analyses further emphasize the immunosuppressive TME in IBC. scRNA‐seq analysis of IBC vs. non‐IBC biopsies showed a reduced proportion of exhausted CD4^+^ T cells (CXCL13^+^CD4^+^ T cells) in the IBC TME, correlating with poor patient outcomes and widespread immune suppression. Spatial analysis confirmed decreased immune cell infiltration (CD45^+^ cells), while CXCL13 overexpression was found to enhance tumor cell death and boost anti‐PD‐1 efficacy [32].

A recent single‐cell transcriptome analysis of three HER2^+^ IBC patient samples identified a novel pleiotrophin (PTN)‐TNF signaling axis that promotes an inflammatory TME. Mechanistically, HER2^+^ IBC cells overexpress PTN, which induces TNF expression in tumor‐infiltrating B cells. This, in turn, promotes necroptosis of endothelial cells, leading to the release of inflammatory cytokines such as IL‐6 and IL‐8, which further promote M2 macrophage polarization and the formation of an immunosuppressive TME, thereby facilitating tumor progression [92].

Finally, AXL signaling also contributes to cytokine‐mediated immune regulation. A recent study elucidated the role of M2 macrophages in promoting IBC cell growth through AXL‐regulated cytokines and chemokines. Specifically, AXL in M2 macrophages regulates the secretion of CCL26, CCL20, and epiregulin via STAT6 signaling, thereby accelerating IBC cell growth and migration [29].

Together, these findings indicate that cytokines and chemokines secreted from both tumor and stromal cells promote an immunosuppressive TME that facilitates IBC immune evasion. Their dual roles in promoting inflammation and immune suppression highlight their potential as therapeutic targets to reprogram the TME and improve the efficacy of immunotherapy in IBC.

### 4.3 Tumor Heterogeneity

Tumor heterogeneity is the morphological and phenotypical diversity among cancer cells, either within a single tumor or between tumors in different patients. Such heterogeneity can affect how cancers grow, spread, and respond to treatment, including immunotherapy [93]. A single‐sample GSEA analysis of various IBC gene signatures revealed that EMT scores in IBC samples exhibited a greater coefficient of variation than those in non‐IBC samples, indicating increased heterogeneity along the EMT spectrum as a defining feature of IBC [94]. A recent scRNA‐seq analysis and spatial transcriptomics study of IBC and non‐IBC biopsies revealed heterogeneity among IBC epithelial cells (Figure 1C). Normal breast tissue is mainly composed of luminal progenitor cells, whereas IBC tumors contain distinct epithelial subsets of luminal A, luminal B, or HER2‐enriched signatures. In one IBC patient sample (luminal B subtype), epithelial clusters are notably enriched and exhibited downregulation of immune and estrogen response pathways, suggesting impaired anti‐tumor immunity and potentially poorer prognosis in ER‐positive cases [32]. Spatial transcriptomics analysis further revealed that tumor‐infiltrating T cells in IBC express significantly lower levels of the chemoattractant CXCL13 than in non‐IBC, and many IBC tumor regions show markedly reduced CD45^+^ immune cell infiltration [32]. These results suggest that distinct microregions within the same tumor may exist in immune‐active or immune‐cold states, potentially leading to different responses to immune surveillance and immunotherapy.

In summary, IBC tumor heterogeneity contributes to immune evasion through phenotypic diversity of tumor cells that evade immune recognition and spatially heterogeneous tumor immune microenvironments that permit immune‐privileged tumor zones. It warrants further investigation to understand the underlying mechanisms, which will help improve immunotherapy for IBC patients.

### 4.4 Tumor Emboli Formation

A key characteristic of IBC is the formation of tumor emboli, cancer cell clusters that spread through the lymphatic vessels in the skin, causing redness and swelling of the breast [95]. Tumor emboli represent a transitional phase between local invasion and distant metastasis, contributing to local recurrence and drug resistance. IBC tumor emboli can form through several mechanisms (Figure 1D). E‐cadherin is overexpressed in about 90% of IBC cases, promoting cohesive cell–cell adhesion and passive collective dissemination as emboli. Proteolytic processing of membrane E‐cadherin (e.g., calpain‐mediated cleavage) and generation of E‐cadherin fragments (E‐cad/NFT1), partly regulated by Rab7, have also been implicated in emboli formation [96, 97]. Altered glycosylation of surface mucins such as MUC1, specifically the reduction of sialyl‐Lewis(x/a) epitopes due to decreased α3/4‐fucosyltransferase activity, impairs E‐selectin binding and may favor emboli dissemination by reducing vascular adhesion [98, 99]. In triple‐negative IBC, GLI1 activation promotes proliferation, motility, tumor growth, and embolization [100].

Tumor emboli may also contribute to immune evasion. They can release molecules that suppress the anti‐tumor immune response and promote the recruitment of immunosuppressive TME components, and shield tumor cells from immune attack [101]. Although the precise mechanism remains unclear, several possibilities have been proposed. For example, cohesive emboli may form a physical barrier that limits lymphocyte and natural killer cell infiltration, reducing antigen exposure. In addition, anti‐apoptotic signaling (NF‐κB, XIAP) can confer resistance to cytotoxic effector mechanisms, while hypoxia and the presence of M2 macrophages and MSCs can further foster immunosuppression through TGF‐β, IL‐10, and other chemokines [102, 103, 104]. These combined mechanisms may allow tumor emboli to persist and disseminate while evading immune surveillance. While direct evidence regarding the exact pathways of tumor emboli formation is still lacking, elucidating this mechanism and its immunosuppressive effects will be critical for developing effective therapeutic strategies.

### 4.5 Summary

IBC employs multifaceted mechanisms of immune evasion that collectively sustain an immunosuppressive TME and confer resistance to immunotherapy. IBC tumors frequently overexpress immune checkpoint molecules, including PD‐L1, LAG‐3, CTLA‐4, and TIM‐3, leading to profound T‐cell exhaustion and suppression of cytotoxic activity. Tumor‐intrinsic oncogenic signaling, particularly through EGFR, XIAP, RhoC GTPase, AXL, and downstream NF‐κB, JAK/STAT, and MAPK pathways, reprograms the surrounding stroma by inducing pro‐tumor cytokines and chemokines, such as CCL2, CCL20, IL‐6, and IL‐8, which recruit M2‐like macrophages, Tregs, and other immunosuppressive cells. The resulting cytokine loops reinforce immune exclusion, angiogenesis, and EMT. Tumor heterogeneity further amplifies this effect by creating spatially distinct immune‐active and immune‐cold regions, while cohesive tumor emboli physically and functionally shield tumor cells from immune attack. Together, these features produce a dynamic yet highly suppressive ecosystem that undermines ICIs.

## 5 Conclusion and Future Directions

IBC remains one of the deadliest and biologically complex forms of breast cancer, defined by rapid progression, high metastatic potential, and profound resistance to conventional therapies. Mechanistic and translational studies have illuminated how tumor‐intrinsic oncogenic signaling through EGFR, AXL, RhoC GTPase, XIAP, and the IL‐6/IL‐8 cytokine axis coordinates with stromal and immune components to create an immunosuppressive, macrophage‐dominant TME. This intricate network underlies immune evasion and limits the efficacy of ICI in IBC.

Transforming the IBC TME from immune‐cold to immune‐active has therefore become a central therapeutic goal. Promising avenues include strategies that repolarize macrophages, restore DC function, and normalize vasculature to facilitate T‐cell recruitment. Combination regimens integrating ICIs with ADCs, cytokine modulators, radiotherapy, or targeted inhibitors for EGFR and AXL are under active investigation to overcome immune resistance.

It is worth noting that the lack of immunocompetent syngeneic IBC mouse models remains a major limitation in preclinical IBC research. This limitation restricts the study of tumor‐immune interactions and the development of novel combination strategies to enhance immunotherapy efficacy. Humanized mouse models partially address this issue by enabling the investigation of both tumor and TME crosstalk and immunotherapy responses [82]. However, these models still have limitations, such as incomplete HLA matching. The development of syngeneic IBC mouse models and next‐generation humanized IBC models with complete HLA matching will be critical for identifying effective immunotherapy targets and combination strategies.

Data presented at ESMO 2025 and ASCO 2025 have highlighted the potential of integrating ADCs, including T‐DXd and datopotamab deruxtecan (Dato‐DXd), into the neoadjuvant and adjuvant settings, which have achieved unprecedented responses in HER2‐positive and HER2‐low breast cancers. For IBC, these breakthroughs open a new therapeutic paradigm, in which ADCs function not only as cytotoxic agents but also as immunologic primers that can reshape the TME and enhance IBC sensitivity to ICIs. Early‐phase studies testing T‐DXd plus durvalumab in HER2‐positive IBC (NCT05795101) and Dato‐DXd or sacituzumab govitecan plus pembrolizumab in HER2‐low or triple‐negative disease exemplify this evolving strategy. The next generation of combination modalities that link ADC‐induced immunogenic cell death with macrophage or cytokine modulation, radiotherapy, or local immune activation represents a rational path to overcome therapeutic resistance.

Looking forward, precision strategies that combine ICI‐based therapy with immunomodulatory agents, including c‐MET inhibitors [105] or localized immune activation through stereotactic radiotherapy and immune injections [106], hold particular promise. Embedding single‐cell and spatial profiling into adaptive, biomarker‐driven clinical trial designs will be critical for identifying predictive markers and optimizing patient selection. The establishment of a dedicated IBC diagnostic code further facilitates accurate case identification, longitudinal tracking, and multi‐institutional collaboration, paving the way for statistically robust, IBC‐specific clinical trials.

Collectively, these advances are reshaping the therapeutic landscape of IBC. Sustained investment in mechanistic research and rationally designed immunotherapy trials will be essential to transform this immune‐refractory disease into one responsive to durable, precision‐guided immune control, and to ensure IBC remains at the forefront of the next era of breast cancer innovation.

## Conflicts of Interest

NTU holds consulting roles with AstraZeneca, Bayer, Pfizer, Gilead Sciences, Chugai Pharmaceutical, Daiichi Sankyo, Eisai Co., OBI Pharma, OncoCyte, Ourotech, DBA Pear Bio, Phoenix Molecular Designs, CARNA Biosciences, ChemDiv, DualityBio, Merck, Carisma Therapeutics, and Eli Lilly. He also has research agreements in place withOBI Pharma.

## Data Availability

Data sharing not applicable to this article as no datasets were generated or analysed during the current study.
